# Salvage Radiosurgery for Recurrent Cardiac Sarcoma: A Case Report

**DOI:** 10.7759/cureus.44990

**Published:** 2023-09-10

**Authors:** Sophia N Shah, Sohan S Shah, Martha Hosford-Skapof, Sunjay A Shah

**Affiliations:** 1 Radiation Oncology, Christiana Care Health System, Newark, USA; 2 Hematology Oncology, Christiana Care Health System, Newark, USA

**Keywords:** cardiac sarcoma, 3d conformal radiation therapy, salvage radiosurgery, single fraction stereotactic radiosurgery, palliative radiation therapy

## Abstract

Primary cardiac sarcoma is a rare malignant tumor that arises from the cardiac myocardium. Surgical resection is the standard of care, and median survival ranges from 6 to 12 months. The role of salvage chemotherapy and radiation is not well defined. A 53-year-old female presented with acute congestive heart failure and underwent complete surgical resection of an undifferentiated pleomorphic sarcoma of the left atrium, followed by six cycles of adjuvant doxorubicin/hydroxydaunorubicin and ifosfamide. An MRI scan demonstrated an asymptomatic, 24 mm, recurrent atrial mass. The patient was treated with frameless robotic radiation therapy over three weeks. The tumor was treated with a dose of 72 Gy in 15 fractions to the 84% isodose line. A repeat cardiac MRI at four weeks showed in-field local progression with greater protrusion into the left atrium and invasion of the left ventricle. The patient therefore elected to proceed with salvage single-fraction frameless robotic radiosurgery. 25 Gy in one fraction was prescribed to the 76% isodose line. She tolerated treatment well without any acute toxicity and was subsequently treated with a variety of chemotherapy regimens, including tyrosine kinase inhibitors (TKIs) and immunotherapy. Unfortunately, the patient relapsed with metastases in the spine and pelvis. She underwent palliative radiation therapy at multiple bony sites with a partial response. She resumed chemotherapy treatment with TKIs but passed away due to septic shock without evidence of local failure. Fractionated SBRT was ineffective at controlling our patient's cardiac sarcoma. Our patient demonstrated local control of disease at 12 months after salvage of 25 Gy in one fraction of radiosurgery without any evidence of cardiac toxicity. High-dose single-fraction radiosurgery is a reasonable palliative option for long-term local control of unresectable cardiac sarcomas.

## Introduction

Primary cardiac sarcoma is a highly malignant tumor that arises from the cardiac myocardium with invariably rapid local progression and distant metastases. The median survival has been reported in one series to be only six weeks. Cardiac sarcomas are rare and found in less than 0.03% of autopsy specimens [1], presumably because cardiac myocytes differentiate early in an individual’s life. As cardiac sarcomas are so rare, no prospective trials are available to guide treatment decisions. Surgical resection is the standard of care, and median survival after complete surgical resection is typically six to twelve months. Unfortunately, a microscopically complete or R0 resection is only achieved in about 12% of cases [2], and salvage surgery is almost always ineffective. The role of adjuvant chemotherapy and radiation is not well defined. Radiation is rarely utilized because of its lack of efficacy and potential cardiac toxicity. Stereotactic ablative body radiation therapy (SBRT) is a highly conformal radiation technique with the potential to deliver ablative doses of radiation to the tumor and minimize radiation to substructures of the heart and adjacent critical tissues such as the esophagus and proximal bronchial tree. The CyberKnife device is a method of delivering SABR that utilizes real-time tracking of either implanted fiducials or the spine. Moderate-dose SABR using a CyberKnife device for cardiac sarcoma has been presented in two case reports [3,4]. High-dose SBRT has generated tremendous interest as a possible treatment for ventricular tachycardia with acceptably low toxicity at intermediate follow-up [5]. We therefore elected to administer high-dose SBRT to a patient with multiple recurrent cardiac sarcomas with no other treatment options.

The abstract of this paper was previously presented as a poster at the 2021 Multidisciplinary Thoracic Cancer Symposium on 12/02/2021.

## Case presentation

**Figure 1:**
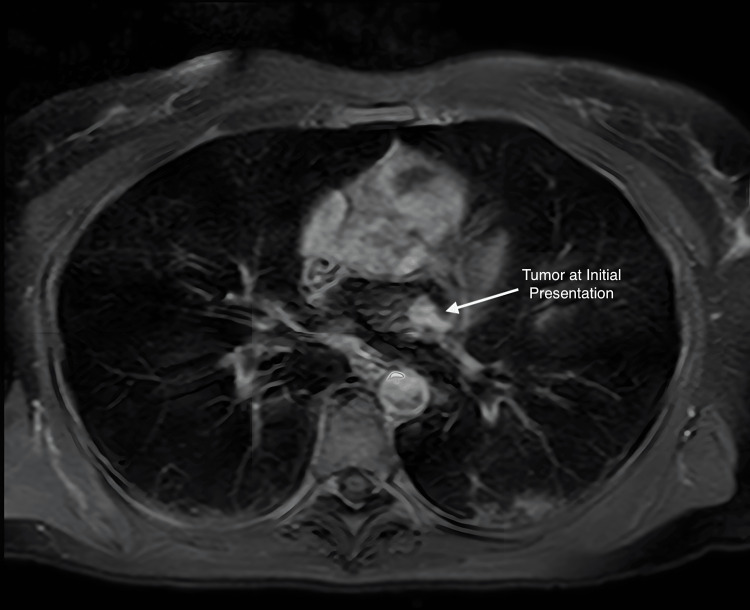
Axial MRI scan of the heart demonstrating a 1.3 cm × 1.2 cm mass in the left atrium along the supero-lateral wall.

**Figure 2:**
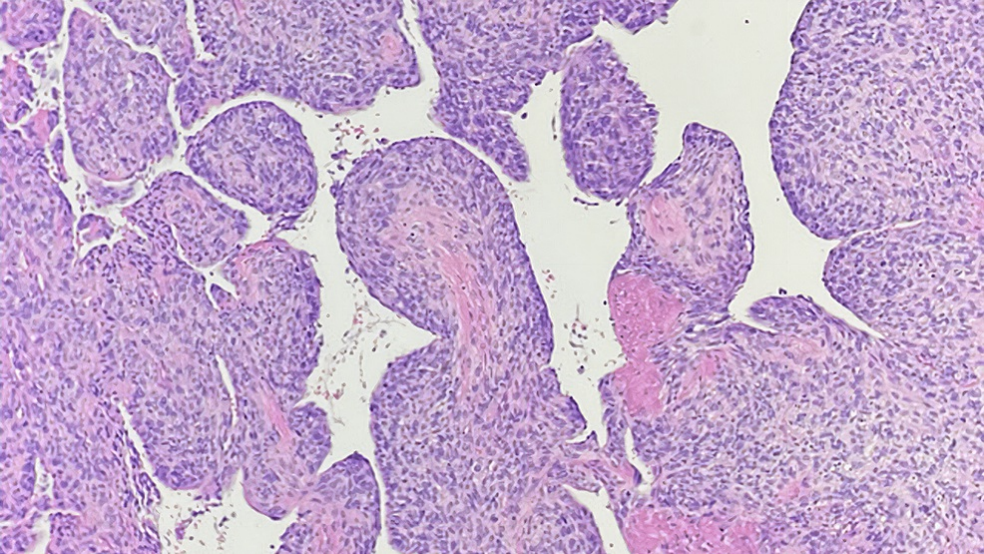
Low power slide demonstrated pleomorphic high-grade undifferentiated cardiac sarcoma.

**Figure 3:**
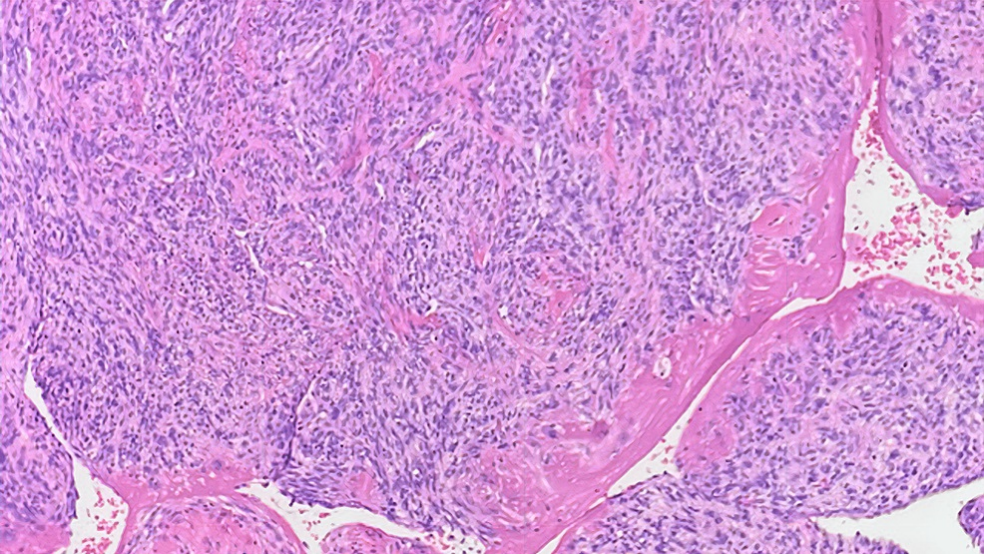
High power view of pleomorphic high-grade undifferentiated cardiac sarcoma.

**Figure 4:**
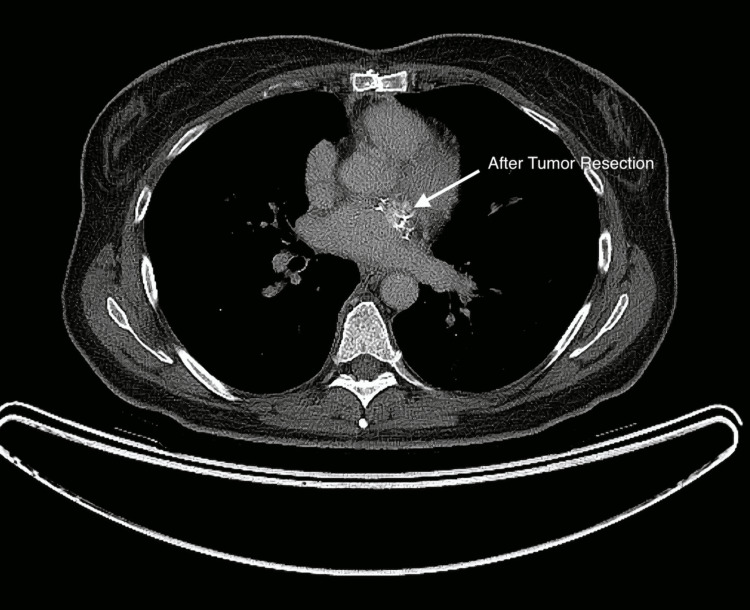
Postoperative CT scan on 12/27/2017 demonstrating no residual disease.

A 53-year-old previously healthy female presented in October 2017 to her family physician with a new systolic murmur on a routine physical exam. She was referred to a cardiologist, who performed an echocardiogram, revealing severe mitral regurgitation. An MRI scan of the heart (Figure 1) revealed a 1.3 cm × 1.2 cm mass in her left atrium along the superolateral wall, and atrial myxoma was suspected. She presented acutely in November 2017 with congestive heart failure. An emergent thoracotomy and atrial tumor resection were performed, along with the closure of the left atrial wall defect with autologous pericardium and the replacement of the mitral valve with a biologic valve. An exophytic tumor was removed in a piecemeal fashion without penetration of the interior of the atria. The final pathology demonstrated pleomorphic high-grade cardiac undifferentiated sarcoma with negative microscopic margins (Figures 2-3). Fluorescence in situ hybridization (FISH) for MDM2 was negative. A postoperative cardiac CT on 12/27/2017 showed no residual disease (Figure 4). Due to the high risk of recurrence, she elected to undergo six cycles of adjuvant chemotherapy with adriamycin and ifosfamide. The first cycle was complicated by severe neutropenia as well as rectal bleeding due to micro-perforation of the cecum. The patient otherwise did well and was carefully followed.

**Figure 5:**
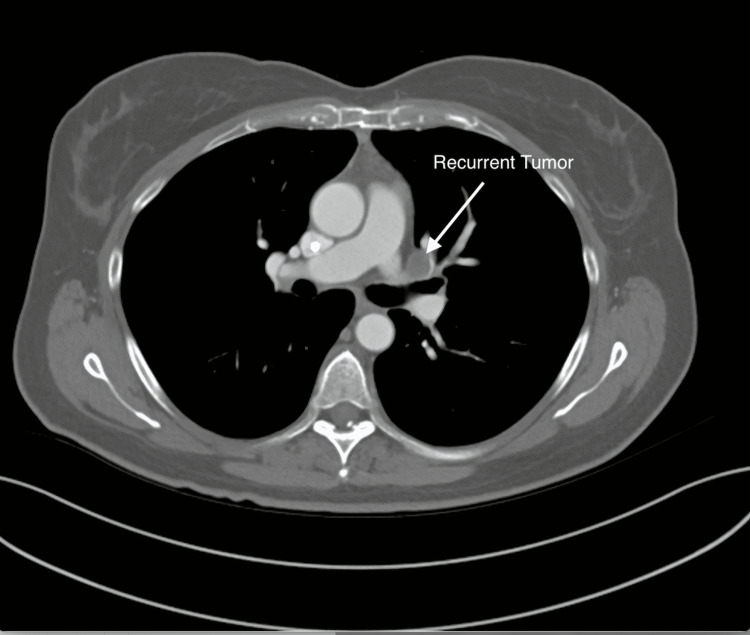
Follow-up CT scan on 3/5/19, demonstrating a new 1.4 cm filling defect in the left upper lobe pulmonary vein.

**Figure 6:**
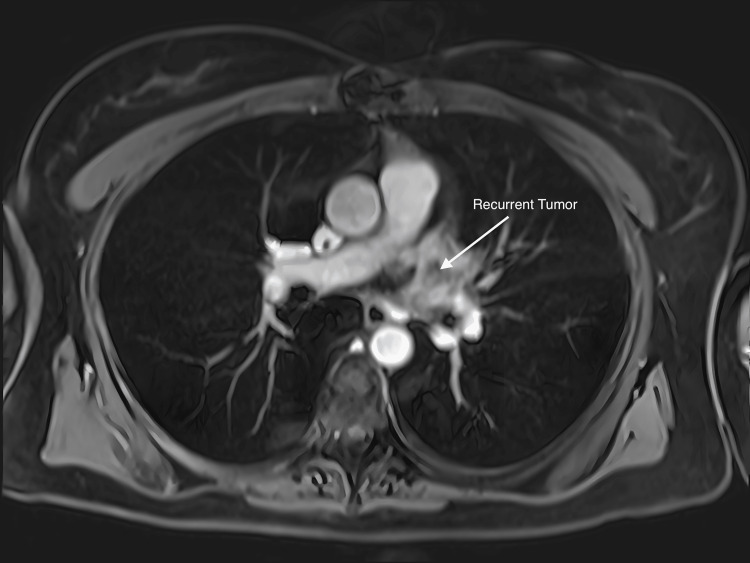
Axial MRI scan on 3/25/19 demonstrating a recurrent atrial mass.

**Figure 7:**
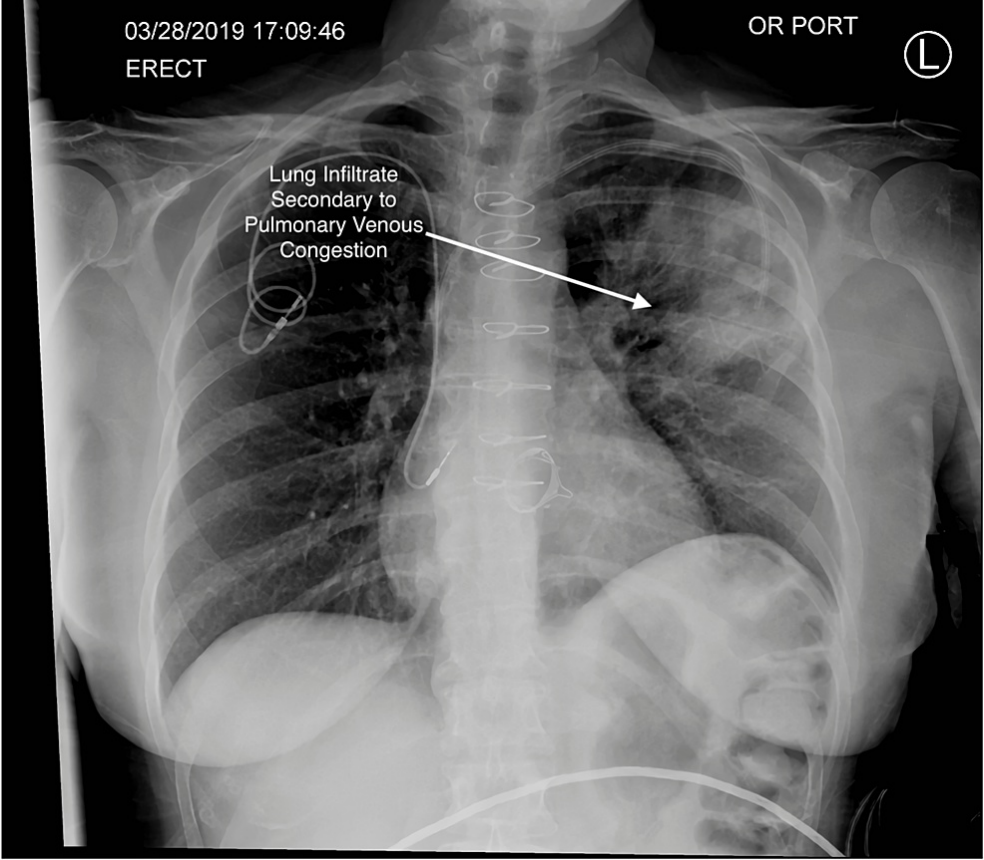
Chest X-ray demonstrating new parenchymal opacities in the left upper lobe secondary to pulmonary venous obstruction.

A transthoracic echocardiogram on 3/3/2019, revealed normal left ventricular systolic function with an EF of 55% and mild to moderate tricuspid regurgitation. A routine follow-up CT scan on 3/5/19 (Figure 5) demonstrated a new 1.4 cm filling defect in the left upper lobe pulmonary vein. A subsequent PET scan on 3/14/19 demonstrated intense hypermetabolic activity of the atrial lesion with a standardized uptake value (SUV) of 17.5. An MRI scan on 3/25/19 (Figure 6) demonstrated a 24 mm atrial mass. The lesion extended to the level of the left upper lobe pulmonary vein centrally, just prior to its confluence with the left atrium and left inferior pulmonary vein. The patient developed a new-onset non-productive cough, and a chest X-ray (Figure 7) showed new pulmonary parenchymal opacities in the left upper lobe secondary to pulmonary venous obstruction. She was evaluated at multiple tertiary medical centers for recurrent cardiac sarcoma, and they felt that she was inoperable. The patient was referred to radiation oncology for evaluation. After a full discussion of options, the patient elected to proceed with salvage CyberKnife stereotactic body radiosurgery.

**Figure 8:**
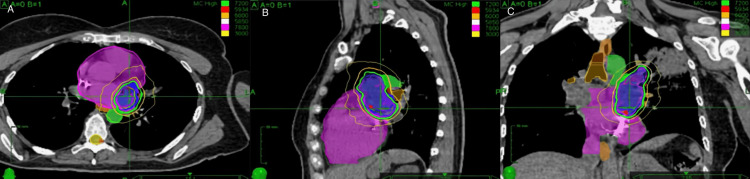
Hypofractionated CyberKnife radiation plan. A dose of 72 Gy in 15 fractions was prescribed to the 84% isodose line. (a) Radiation plan in the axial plane; (b) radiation plan in the sagittal plane; (c) radiation plan in the coronal plane.

**Figure 9:**
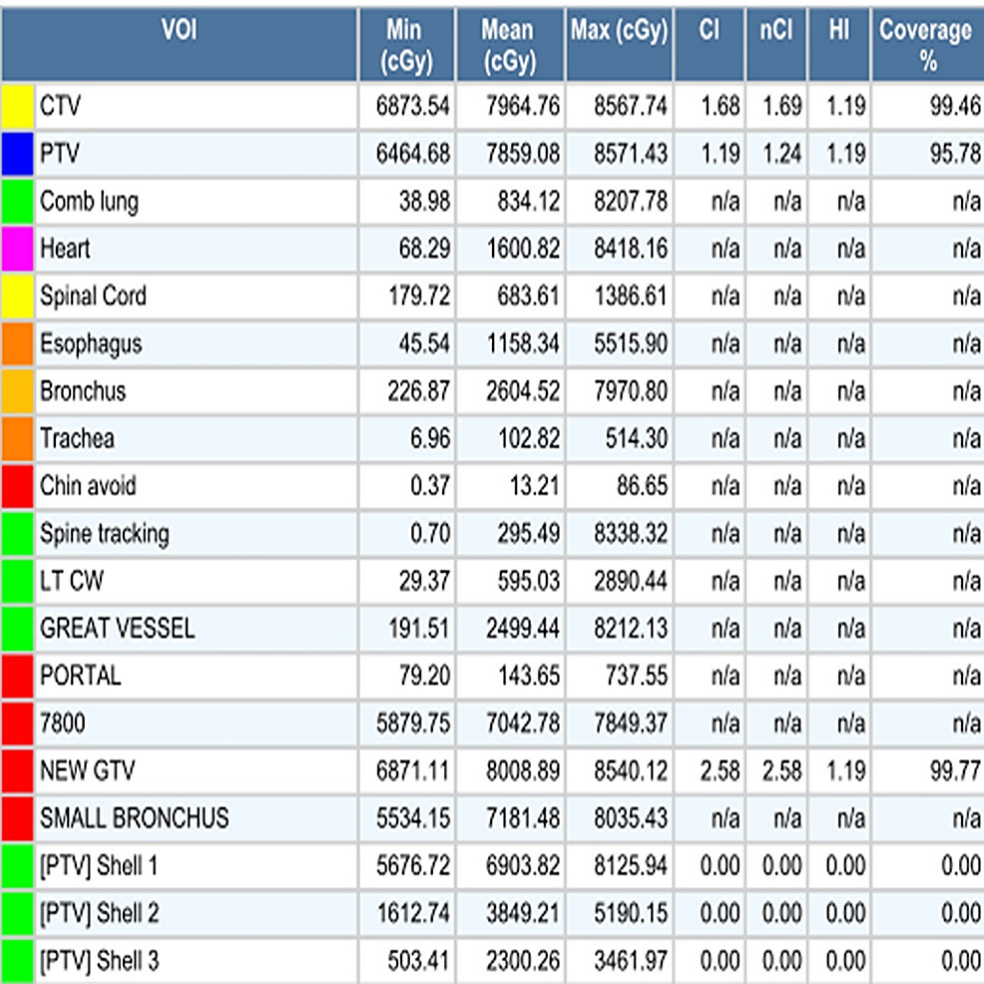
Dose volume table for the first hypofractionated CyberKnife radiation plan.

**Figure 10:**
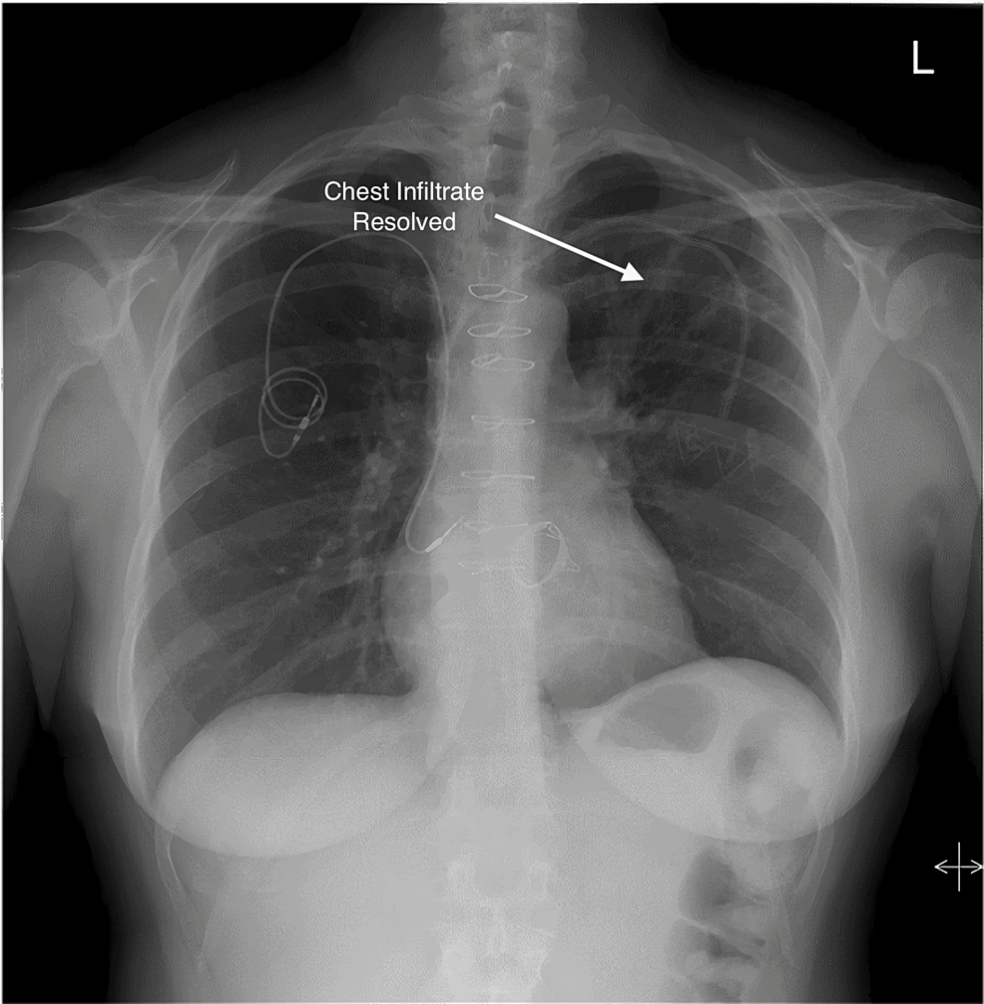
Chest X-ray demonstrating resolution of the left upper lobe pulmonary infiltrate.

CT, MRI, and PET imaging modalities were fused to determine the target volume. An interventional cardiologist placed a Medtronic defibrillator lead in the interatrial septum to track motion using the CyberKnife synchrony software. Unfortunately, the CyberKnife Synchrony software could not track the fiducial due to the cardiac motion. Instead, 4D-CT planning was used to generate the internal target volume (ITV), which was also the planning target volume (PTV). The tumor was prescribed a dose of 72 Gy in 15 fractions to the 84% isodose line between 4/4/2/19 and 4/24/2019. The conformality index was 1.19, and the plan utilized 142 total beams to achieve acceptable maximal doses to the main stem bronchus and esophagus (Figures 8-9). The patient tolerated treatment well, with no acute toxicities. A chest X-ray (Figure 10) at the end of treatment demonstrated resolution of the left upper lobe pulmonary infiltrate.

**Figure 11:**
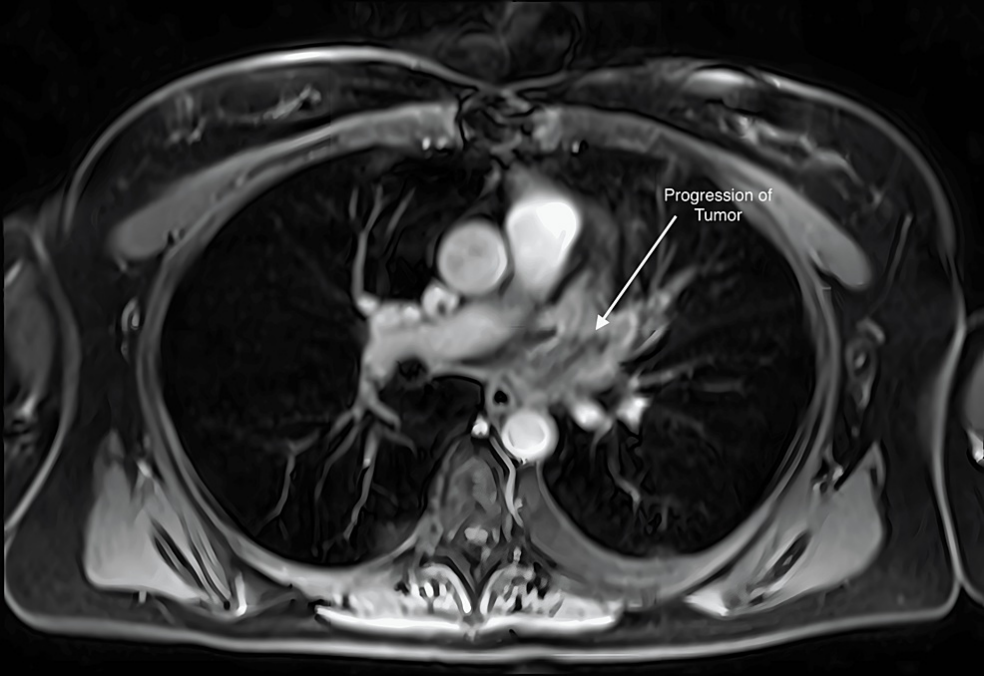
Cardiac MRI at four weeks demonstrating greater protrusion into the left atrium and new partial impediment of the inflow of the left lower pulmonary vein. The component of the mass at the level of the left atrium measured 4.0 cm × 2.7 cm.

**Figure 12:**
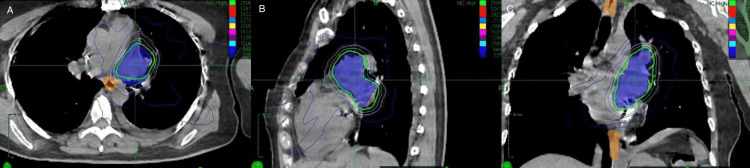
Single fraction CyberKnife radiation plan. 25 Gy in one fraction was prescribed to the 76% isodose line. (a) Radiation plan in the axial plane; (b) radiation plan in the sagittal plane; (c) radiation plan in the coronal plane.

**Figure 13:**
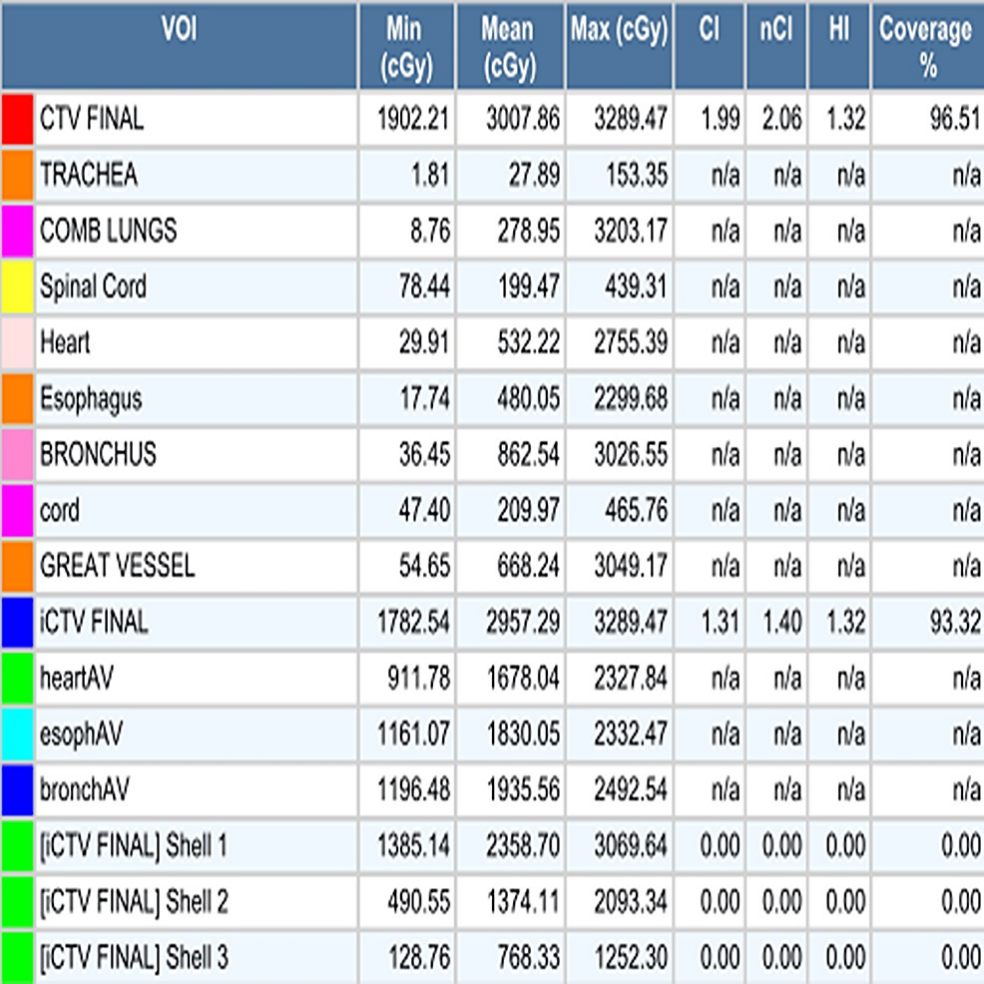
Dose volume table for the salvage single fraction CyberKnife radiation plan.

A repeat cardiac MRI (Figure 11) at four weeks unfortunately demonstrated clear progression of the lesion. There was greater protrusion into the left atrium and a new partial impediment to the inflow of the left lower pulmonary vein. The component of the mass at the level of the left atrium increased from 2.5 cm × 2.0 cm to 4.0 cm × 2.7 cm, and the volume was 60.2 ccs. We had an in-depth discussion with the patient and her husband about various options in the management of her recurrent left atrial sarcoma. She elected to retreat the tumor with salvage high-dose SBRT again with the CyberKnife device. 25 Gy in one fraction was prescribed to the 76% isodose line on 6/13/2019. The conformality index was 1.31, and the plan utilized 152 total beams. MRI and PET fusion were used with 4D-CT planning to generate the iGTV and PTV (Figures 12-13). She tolerated treatment well without any acute or subacute toxicity. Multiple three-week cycles of PD1 inhibitors were administered to the patient following the treatment.

**Figure 14:**
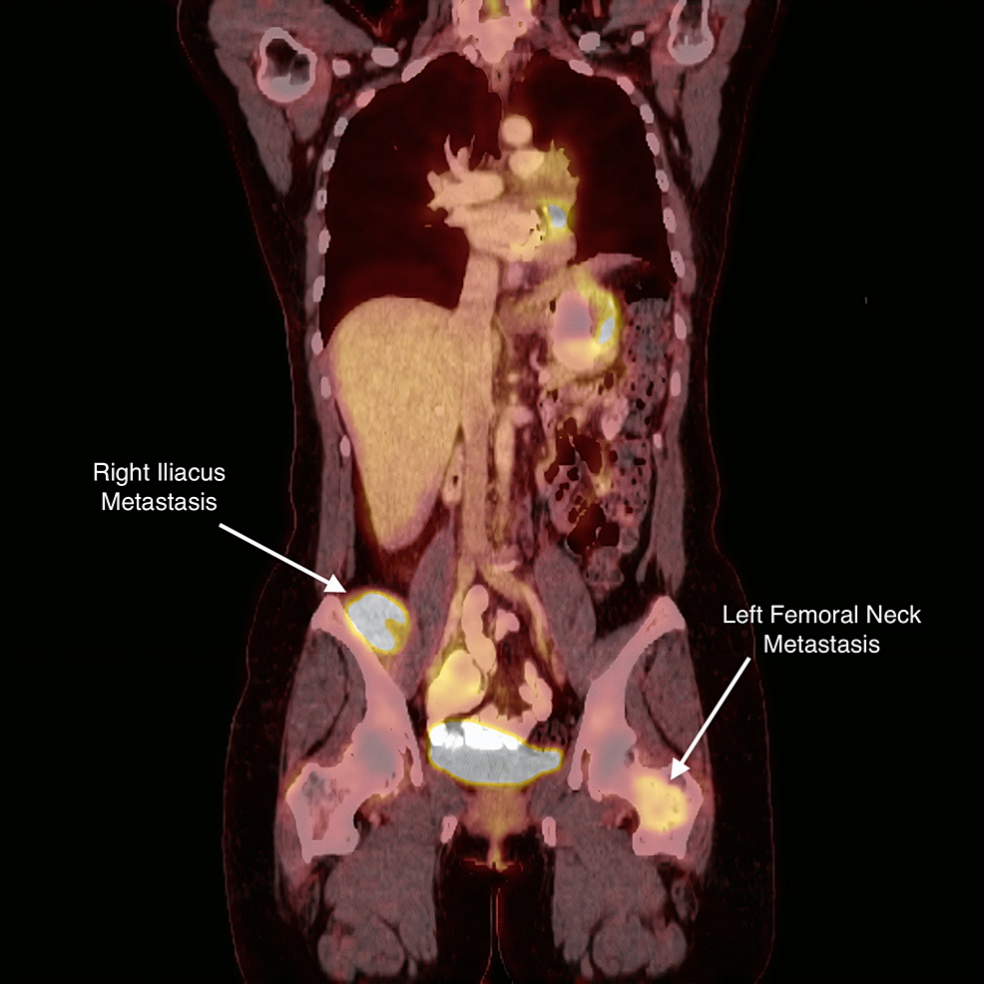
Repeat PET CT scan, conducted on 1/13/2020, demonstrating multiple new hypermetabolic osseous lesions involving the iliacus muscle and femoral neck. No demonstrable evidence of local recurrence within the heart.

Unfortunately, the patient suffered an isolated oligometastatic relapse in December 2019 with a large metastasis of the anterior 2/3rds of the T11 vertebral body. She underwent salvage SBRT at this site on 12/19/2019. The tumor was treated with a dose of 25 Gy in one fraction. She tolerated treatment well without any toxicity. Unfortunately, a repeat PET CT scan on 1/13/20 (Figure 14) demonstrated multiple new hypermetabolic osseous lesions. The largest was in the right pelvic sidewall, involving the iliacus muscle. There was no evidence of local recurrence within the heart. There was increased uptake along the greater curvature of the stomach as well as the ascending colon, which was suspicious for metastatic disease. The T11 vertebral body was stable. There were new lesions in the right L4/L5 vertebral body, the right posterior iliac bone, the right posterior acetabulum, as well as the left femoral neck. The patient was treated at each of these sites with a palliative dose of 25 Gy in five fractions at 5 Gy per fraction. The pain at her four sites treated with radiation decreased by at least 90%. She then started salvaging Votrient (Table 1 shows a summary of radiation treatments).

**Table 1:** Radiation treatments administered.

Site	Start	End	Dose	Fractions
1. CK Lt Heart	4/4/19	4/24/19	72 Gy	15
2. CK LT Heart	6/13/19	6/13/19	25 Gy	1
3. CK T11 Spine	12/19/19	12/19/19	25 Gy	1
4. RT Pelvis	1/27/20	1/31/20	25 Gy	5
5. LT Femoral Neck	1/27/20	1/31/20	25 Gy	5
6. LT Ankle	1/27/20	1/31/20	25 Gy	5
7. TSpine (t10-12)	2/3/20	2/7/20	22.5 Gy	5

**Figure 15:**
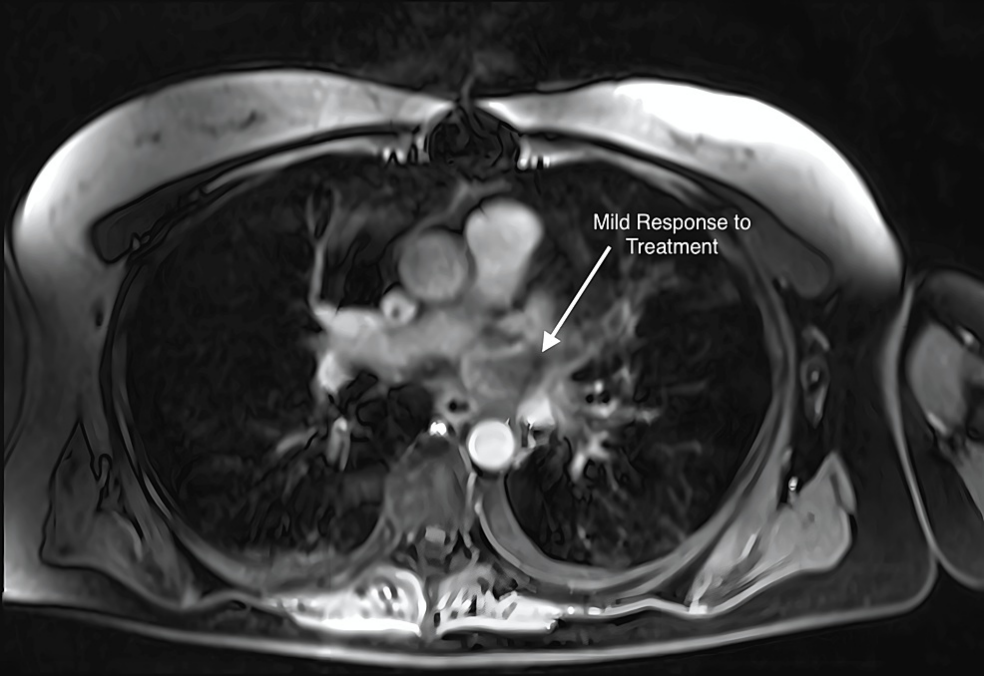
Axial MRI performed 3/20/2020 demonstrating partial response of the cardiac sarcoma.

When last seen on 3/20/2020, she continued to have an excellent palliative response to XRT at multiple sites, along with a partial response to the cardiac tumor (Figure 15). She resumed chemotherapy treatment with tyrosine kinase inhibitors (TKIs) but was admitted to the hospital in June with altered mental status secondary to toxic metabolic encephalopathy. She expired due to septic shock, DIC, and acute respiratory failure 16 months after salvaging SBRT. There was no evidence of local cardiac recurrence or cardiac toxicity.

## Discussion

Primary cardiac sarcomas are extremely rare tumors with a poor prognosis, even with R0 surgical resection. Because cardiac sarcomas are so rare, there are few studies to determine which treatment option is most effective [1]. Treatments for cardiac sarcoma depend on a variety of factors, such as one’s age, medical history, and the extent of the disease. The roles of chemotherapy and radiation therapy are not well defined. A retrospective review from the Cleveland Clinic suggested that multimodality treatment with surgery and radiation is more effective than single-modality treatment alone [6]. Cardiac transplantation has recently emerged as a treatment option [7].

With improvements in technology in recent years, stereotactic radiosurgery has become another option for thoracic cancer treatment, and it has been shown to be effective in treating localized lung and spine tumors. Radiation treatment is rarely used because of the heart’s sensitivity to radiation compared to other organs. Traditionally, 22 Gy was considered to be the maximum dose for single-dose SBRT for the heart [8]. However, Robinson et al. reported that ventricular tachycardia was treated by a 25 Gy dose of radiation to the heart by SBRT, and no acute toxicity was observed. This suggested that the heart could be treated safely with higher doses of radiation than previously realized [5,9].

In the first treatment of radiation to the heart from 4/4/19 to 4/24/19, the dose was 72 Gy in 15 fractions over three weeks. This fractionated dose was selected due to the tumor's immediate proximity to the main stem branches and the esophagus. However, cardiac MRI demonstrated a doubling of the tumor volume within four weeks with secondary pulmonary venous congestion, suggesting that high-dose fractionated radiation was ineffective. In the salvage treatment of SBRT on 6/13/19, 25 Gy in a single fraction was utilized. Surprisingly, after this treatment, the tumor did not recur locally, although the patient died of distant metastases at 16 months.

There are two case reports in the literature on CyberKnife treatment for cardiac sarcomas. In a 2008 report from Stanford University, 33 Gy in three fractions was prescribed to the 79% isodose line, and the patient tolerated the treatment well with no acute adverse side effects. A post-treatment CT angiogram displayed a decrease in the pulmonary artery tumor, but the patient expired 10 weeks after treatment due to diffuse multifocal lung metastases [4]. In an Italian study, three patients had SBRT for cardiac lesions. Two patients had recurrent cardiac angiosarcomas previously treated with radiation, while one patient had a cardiac metastasis from melanoma. They were treated with fiducial-guided robotic radiotherapy with CyberKnife. 24 Gy in three fractions (80% isodose) and 30 Gy in five fractions (80% isodose) were given. Six months after treatment, the cardiac MRI confirmed the absence of local progression in all the cases [3].

In the first radiation treatment, we attempted to minimize the planning target volume by using synchrony fiducial tracking, as per the report of Bonomo et al. [3]. Although we were able to track a cardiac defibrillator lead in a dynamic respiratory phantom, we could not utilize the lead as a fiducial in the patient due to cardiac motion. The patient was therefore treated using spine tracking with a respiratory motion expansion as determined on a 4D-CT scan. The 4D-CT planning resulted in a 50% greater target volume compared to the volume that would have been treated utilizing fiducial tracking. The patient did not have any noticeable acute or late toxicity from her courses of radiation, suggesting that the heart can withstand higher amounts of radiation than previously assumed.

## Conclusions

Our patient demonstrated local control of multiple recurrent cardiac sarcomas at 16 months without evidence of acute or late cardiac toxicity. Our case demonstrates several important points. Even high-dose fractionated radiation therapy was unsuccessful in controlling our case of cardiac sarcoma. On the other hand, high-dose salvage SBRT of 25 Gy × 1 resulted in long-term local control without acute or late cardiac toxicity, confirming the report from Washington University. This is the longest-reported local control in the literature using any radiation modality. 4D-CT is sufficient to determine the planning target volume, even in the absence of fiducial tracking. A likely factor in success in this case was the availability of expertise in cardiac MRI interpretation to accurately determine the target volume and response to treatment. Our case supports the concept of SBRT as a more effective modality than conventional radiation therapy for the management of cardiac sarcomas and should be considered as an option for the treatment of inoperable and recurrent cases.
